# The role of family quality of life in the caregivers of autistic adolescents receiving treatment for daily living skills

**DOI:** 10.1016/j.reia.2025.202660

**Published:** 2025-07-11

**Authors:** Amie Duncan, Lauren Tadevich

**Affiliations:** a Cincinnati Children’s Hospital Medical Center, Department of Pediatrics, Cincinnati, OH, USA; b Department of Pediatrics, University of Cincinnati College of Medicine, Cincinnati, OH, USA

**Keywords:** Autism, Family quality of life, Daily living skills, Adolescence, Intervention, Adaptive, Behavior

## Abstract

Adolescence may be a particularly challenging time for autistic teens and their caregivers. Thus, the current study sought to better understand Family Quality of Life (FQOL) in this population. Further, given the impact that daily living skills (DLS) have on the family system, the current study examined whether a DLS intervention impacted FQOL. Fifty-eight adolescents with autism and their caregivers completed the Surviving and Thriving in the Real World (STRW) intervention and caregivers reported on their FQOL using the *Beach Center Family Quality of Life* (FQOL Scale). Results revealed that caregivers who participated in the STRW intervention endorsed significant positive changes on the Family Interaction (*p* = .02) and Parenting (*p* = .03) subscales and Overall FQOL (*p* = .02) from baseline to post-treatment. Further, higher Emotional Well-Being at baseline was the only FQOL variable that was related to increased changes in DLS from baseline to post-treatment (r = .29, *p* = 03). Results suggest that completion of the STRW intervention relates to significant positive changes in FQOL. Further, results suggest that targeting aspects of FQOL, such as emotional well-being, may lead to improved treatment gains in autistic teens.

## Introduction

### Adolescence in autistic individuals

While autism is a neurodevelopmental disorder characterized by difficulties in social-communication and restricted and repetitive behaviors and interests (American Psychiatric Association, 2013), many autistic adolescents also experience challenges in areas including daily living skills (Glover et al., 2022), executive functioning (Tschida & Yerys, 2022), social interactions with same-aged peers (Kuo et al., 2013), emotion regulation (Conner et al., 2020; Keluskar et al., 2021), and internalizing and externalizing disorders (Malow et al., 2023; McCauley, Elias et al., 2020). Autistic teens must also simultaneously navigate developmental expectations associated with adolescence including identity development, evolving family relationships, and increased independence and autonomy (Arnett, 2014), while also considering how their diagnosis of autism may impact their identity and relationships with others (Botha & Gillespie-Lynch, 2022; Mesa & Hamilton, 2022). Further, while autistic teens may receive essential supports while in high school, they often fall off a “services cliff” after high school graduation such that they may not be eligible for and/or there are limited services available to facilitate a successful adult transition (Laxman et al., 2019). Thus, adolescence may be a particularly challenging time for autistic teens and their caregivers.

### Adult outcomes

Current prevalence estimates indicate that over 60 % of autistic individuals do not have a co-occurring intellectual disability (ID; Shaw et al., 2025). Recent research demonstrates that autistic young adults without ID have poorer outcomes in college, employment, activity involvement, independent living, and overall quality of life compared to same-aged neurotypical peers (Bishop-Fitzpatrick et al., 2018; Clarke et al., 2020; Kirby et al., 2016; Lord et al., 2022; Lord et al., 2020; Lounds Taylor, 2017; McCauley, Elias et al., 2020; Taylor & Seltzer, 2011). Additionally, after graduating high school, autistic individuals have low rates of full-time and part-time employment and 60% of autistic adults without ID continue to live with their family, who often provide significant levels of support (Clarke et al., 2020; Kirby et al., 2016; Lord et al., 2022; Lord et al., 2020; Lounds Taylor, 2017; McCauley, Elias et al., 2020; Pickles et al., 2020). These studies underscore the need to understand how best to intervene in adolescence to increase the likelihood of successful adult outcomes.

### Family quality of life

Another area that undoubtedly impacts autistic individuals during adolescence is their relationships and interactions with their family and the family environment. Families are complex, dynamic, and interconnected systems such that each member and various environmental and sociocultural contexts interact, overlap, and influence one another (Bronfenbrenner, 1979; Turnbull et al., 1984). Family quality of life (FQOL) is defined as “a dynamic sense of well-being for the family, collectively and subjectively defined and informed by its members, in which individual and family-level needs interact” (Zuna et al., 2011, p. 262). FQOL is impacted by how and whether family members enjoy their time together and whether they have opportunities to engage in meaningful experiences together (Park et al., 2003). This concept has been extended to the field of individuals with neurodevelopmental disabilities such that specific FQOL questionnaires assess factors including accessing disability related services, providing care to the autistic individual, and caring for oneself and other family members (Gardiner & Iarocci, 2015).

Much of the research that has been conducted on FQOL in autistic individuals has been done with caregivers of children only or has used samples that consisted of caregivers of both children and adolescents (e.g., Alenazi et al., 2020; Dardas & Ahmad, 2014; Gardiner & Iarocci, 2015; Papadopoulos et al., 2023; Papadopoulos et al., 2024; Vasilopoulou & Nisbet, 2016). One study found that families with a child who has autism or Down syndrome had lower FQOL in the areas of health, financial well-being, and support from others compared to families who had a neurotypical child (Brown et al., 2006). In that same study, families with an autistic child reported lower FQOL than families with a child with Down syndrome. Studies have examined various predictors of FQOL in autistic individuals including child characteristics (e.g., behavior problems, autism severity), family characteristics (e.g., income, spirituality, self-advocacy, family coherence, coping style), and support characteristics (e.g., family-teacher partnerships, social support, adequacy of services provided) (e.g., Davis & Gavidia-Payne, 2009; Hsiao et al., 2017; Poston & Turnbull, 2004; Pozo et al., 2014; Summers et al., 2007; Wang et al., 2004).

A recent study (Hsiao et al., 2017) examined the association between family demographics and parental stress with FQOL in caregivers of autistic children, adolescents, and young adults using the Beach FQOL scale (Hoffman et al., 2006). Caregivers reported the lowest FQOL in Emotional Well-Being (e.g., my family members have the support we need to relieve stress) and Disability Related Supports (e.g., my family member with special needs has support to make progress at home). Results also revealed higher levels of FQOL for caregivers who were male, had a partner or spouse, had at least a bachelor’s degree, had a higher income, and endorsed lower rates of stress.

Adolescence is a particularly critical time to examine FQOL as both autistic teens and their family members are navigating the challenges that naturally arise with the transition from high school to adulthood, yet there have been few studies that have examined FQOL in the caregivers of autistic adolescents. In one study that used the Beach Center FQOL scale in 425 caregivers of 13–21 year-olds with a diagnosis of ID or autism (Boehm et al., 2015), caregivers reported the highest FQOL in Physical and Material Well-Being (e.g., my family has a way to take care of our expenses) and Family Interaction (e.g., my family enjoys spending time together). Caregivers reported the lowest FQOL in Emotional Well-Being, which was similar to the results of the study described above (Hsiao et al., 2017). The study by Boehm and colleagues also found that fewer challenging behaviors, fewer support needs, and strong religious faith predicted higher levels of FQOL. Additional information on the profile of FQOL and predictors of FQOL is needed in the caregivers of autistic adolescents, especially as it relates to developing or tailoring interventions to facilitate successful adult outcomes.

### Role of daily living skills

Daily living skills (DLS), which are the tasks that individuals do to take care of themselves at home, school, and in the community, are often challenging for autistic individuals. Autistic teens without an ID often have DLS that are 6–8 years behind same-aged peers (Duncan & Bishop, 2015) and they may struggle with age-appropriate DLS such as showering, household chores, cooking, laundry, and managing money (Glover et al., 2022). Several studies have demonstrated a relationship between better developed DLS and an increased likelihood of a successful outcome in adulthood for autistic individuals (Clarke et al., 2020; McCauley, Elias et al., 2020; McCauley, Pickles et al., 2020). If a teen has not developed age appropriate DLS, they will likely have challenges transitioning to the adult world of college, work, and independent living. For example, to successfully get to work each day, young adults need to be able to set an alarm to wake up, demonstrate good personal hygiene, get dressed, make breakfast and/or pack a lunch, and get to work (e.g., drive, take the bus). Further, young adults also need to understand how to spend, save, and budget the money earned from their paycheck.

Despite the clear link between DLS and adult outcomes, the Surviving and Thriving in the Real World (STRW) intervention is the only intervention that has demonstrated efficacy in improving DLS in autistic adolescents (Duncan, Liddle et al., 2021). Through a series of studies, autistic teens have gained 2–4 years of DLS after completing the STRW intervention (Duncan, Meinzen-Derr et al., 2021; Duncan et al., 2023; Duncan et al., in press; Duncan et al., 2017), which is a clinically meaningful improvement that narrows the gap between chronological age and actual DLS. The STRW intervention targets age appropriate DLS including hygiene, laundry, cleaning, cooking, and managing money by using individualized evidence-based strategies for teens to incorporate as they work towards their specific DLS goals. Caregivers are an integral component of the intervention as they assist teens with identifying and modifying DLS goals, learning and practicing DLS at home and in the community, and rewarding teens as they make progress towards achieving DLS goals.

While not yet examined, it seems possible that the STRW intervention may impact FQOL because it is implemented as the teen is facing increasing demands with the impending transition from high school to adulthood, which may provide support to both caregivers and teens during an established time of stress (Lounds et al., 2007). For example, during all STRW caregiver group sessions, caregivers are given an opportunity to voice and then problem solve any frustrations or challenges they encounter as they work on building their teen’s DLS, which may decrease their stress level. In support of this, research has shown that problem-focused coping in parents of autistic youth relates to lower parental stress and higher quality of life (Vernhet et al., 2019). Additionally, within STRW, caregivers listen to the experiences of other caregivers, which may normalize some of the emotions they are feeling and build their understanding of how their teen may have similar or different challenges compared to other autistic teens. In support of this, a recent study of a multi-family psychoeducation group intervention that targeted transition related concerns for autistic adolescents found that caregivers highly valued the opportunity to interact with other families (DaWalt et al., 2018). Further, validation from other caregivers of autistic children is often identified as a crucial factor that improves caregiver well-being during group interventions (for review see Catalano et al., 2018). Finally, during the STRW intervention, teens and their caregivers are provided with strategies that may increase their competence, confidence, and independence with achieving current DLS goals and setting goals for building new DLS in the future. It is possible that seeing gains in current DLS goals may positively impact FQOL by decreasing conflicts or anxiety about the teen’s ability to set and achieve future DLS goals that will facilitate their transition to adulthood. This is in line with a growing body of literature demonstrating the positive effects of independence with DLS on well-being for caregivers of autistic children, adolescents, and adults (Fong et al., 2020; Lin et al., 2011; Marsack-Topolewski et al., 2021). Examining whether participating in the STRW intervention positively impacts FQOL would increase our understanding of how the intervention may benefit families beyond improving DLS.

Further, as the STRW intervention involves frequent collaboration and interactions between caregivers and their teens, it would be beneficial to understand if baseline FQOL is related to improvements in DLS after completing the intervention. For example, if a caregiver reports elevated levels of FQOL at the start of the intervention (e.g., increased emotional well-being and parenting confidence), they may be better able to provide the necessary time and energy to their teen by instructing, modeling, and giving constructive feedback on targeted DLS. Understanding the effects of baseline FQOL on DLS improvements after completing the STRW intervention can help us to better understand possible factors that are influencing treatment effectiveness, which can inform future treatment adaptations.

Given that adolescence may be a particularly challenging time for autistic teens and their caregivers and the lack of research on FQOL during this crucial time period, the current study sought to obtain a more comprehensive understanding of FQOL in caregivers of autistic adolescents without an ID and to also explore whether a DLS intervention impacted FQOL. Our specific research questions were: (1) What is the profile on the Beach Center FQOL scale for autistic teens?; (2) What demographic, teen, and parenting characteristics are associated with FQOL?; (3) Does a DLS intervention for autistic adolescents and their caregivers improve FQOL?; and (4) Is FQOL related to change in DLS after receiving the STRW intervention?

## Methods

### Participants

Sixty participants completed the STRW intervention in our two previously published RCTs for autistic adolescents in high school (Duncan et al., 2023; Duncan et al., in press). The inclusion criteria for adolescents for both RCTs was: (1) a diagnosis of autism based on the Autism Diagnostic Observation Schedule, 2nd Edition (ADOS-2; Lord et al., 2012) criteria and/or clinician judgment, (2) full-scale IQ ≥ 70, (3) between 14–21 years of age and enrolled in high school, and (4) presence of a DLS deficit, which was defined as a Vineland Adaptive Behavior Scale, 3rd Edition (VABS-3; Sparrow et al., 2016) DLS domain or subdomain score that was 15 or more points below the full-scale IQ. Exclusion criteria included not being able to attend the majority of the intervention sessions or significant aggressive behavior or mental health issues that would require treatment outside the score of the current interventions being offered. See previously published studies for detailed information on recruitment, randomization, and participant flow.

### Study Procedures

Once participants met inclusion criteria at the in-person eligibility assessment and were enrolled in the RCT, they completed a battery of online questionnaires at the baseline assessment (see Duncan et al., 2023 and Duncan et al., in press for additional information). Participants in the current study then completed the 14-week STRW intervention (either in-person or telehealth). The in-person STRW intervention consisted of weekly caregiver and teen groups facilitated by 2–3 therapists that met concurrently for 90 min. STRW-telehealth, which was implemented due to the COVID-19 pandemic restrictions in March 2020, consisted of weekly caregiver groups (90 min) and weekly caregiver/teen dyad sessions (60 min) delivered through videoconferencing by 2–3 therapists. All sessions were facilitated by therapists (e.g., psychologist, clinical psychology post-doctoral fellow) who received training in the STRW intervention and attended weekly supervision sessions with other STRW therapists (see Duncan et al., 2021 for additional information on intervention personnel). The content for both in-person STRW and STRW-telehealth was identical. Approximately 16 participants (27.6%) received in-person STRW and 42 participants (72.4%) received STRW-telehealth. Within 1 month of completing the STRW intervention, caregiver participants completed the battery of online questionnaires at the post-treatment assessment. This study was approved by the institutional review board for human subjects at Cincinnati Children’s Hospital Medical Center. Informed consent was obtained at the baseline assessment from the caregiver and adolescent prior to data collection.

### Surviving and Thriving in the Real World (STRW) intervention

The STRW intervention consists of 14 sessions that target age appropriate DLS (e.g., hygiene, laundry, cleaning, cooking, grocery shopping, saving and spending money) in autistic adolescents without an ID (Duncan et al., 2021). An essential treatment component of STRW is that the caregiver and teen collaboratively identify and define DLS goals that the teen will work towards using a DLS contract. Considerable time is spent in each session discussing the DLS contract and how it can be modified to consider the teen (e.g., strengths, challenges, learning preferences, commitment, preferred rewards) and the caregiver (e.g., time, energy, commitment, family and household dynamics). The DLS contract also incorporates evidence-based strategies (e.g., recurring phone alarm to wake up, task analysis of laundry steps, video modeling to learn how to make scrambled eggs) to increase the likelihood of meeting DLS goals.

While no components of the STRW intervention directly target the nature and quality of the relationship between the caregiver and teen, it is possible that elements of the intervention may have a positive effect on the relationship by decreasing stress for the caregiver. For example, elements such as getting emotional support from other caregivers, getting individualized problem solving assistance from the therapist, and learning specific strategies to build DLS may decrease caregiver stress, increase adolescent autonomy, and positively impact the parent-child relationship.

### Beach center Family Quality of Life Scale (FQOL Scale)

At baseline and the post-treatment assessment, The Beach Center Family Quality of Life (FQOL) Scale (Hoffman et al., 2006) was completed to obtain information about the caregiver’s perception of family quality of life. The FQOL Scale consists of 25 items that are rated on a 5-point scale from Very Dissatisfied to Very Satisfied. The FQOL Scale contains 5 subscales with 4–6 items each: (1) Family Interaction assesses relationships among and between family members (e.g., “My family solves problems together”); (2) Parenting assesses the activities that families engage in to facilitate their child’s development (e.g., “Family members help the children learn to be independent”); (3) Emotional Well-Being measures the perception of stress and availability of support (e.g., “My family has the support we need to relieve stress”); (4) Physical/Material Well-Being assesses basic physical needs such as medical care and transportation (e. g., “My family has a way to take care of our expenses”); and (5) Disability Related Support measures supports at home, school, and in the community (e.g., “My family member with special needs has support to make progress at home”). The scale’s instructions asks caregivers to define family members as anyone who is part of their family or the individuals who have supported and cared for each other on a regular basis. The FQOL Scale has been shown to be reliable and valid (see Hoffman et al., 2006). As recommended by the developers of the FQOL Scale, the raw score for each of the 5 subscales was calculated and divided by the number of items on the subscale to obtain an average score. A Total FQOL Scale average score was calculated by summing the 5 subscale raw scores and dividing by the number of total items. Higher scores indicate increased levels of FQOL.

### Demographic questionnaire

At baseline, all caregivers completed a demographics questionnaire about the adolescent’s age, sex at birth, race, and ethnicity and caregiver education and income.

### Cognitive abilities

At the in-person eligibility assessment, adolescents completed cognitive testing to obtain the full-scale IQ standard score.

### Autism symptomatology

#### Autism Diagnostic Observation Schedule, 2^nd^ Edition (ADOS-2).

At the in-person eligibility assessment, all adolescents completed the ADOS-2 (Lord et al., 2012) to confirm a diagnosis of autism. The Comparison Score for the overall total score on the ADOS-2 protocol ranges from 1–10 with a higher score indicating increased autism symptom frequency and severity.

#### Social Responsiveness Scale-2^nd^ Edition (SRS-2).

At baseline, all caregivers completed the SRS-2 (Constantino, 2012) to obtain information about the adolescent’s autism symptoms. The SRS-2 consists of 65-items and yields five subscale scores in the areas of Social Awareness, Social Cognition, Social Communication, Social Motivation, Restricted Interests and Repetitive Behavior, and Total. All areas yield T-scores (M=50, SD=10).

#### Vineland Adaptive Behavior Scales, 3^rd^ Edition (VABS-3).

At the in-person eligibility assessment and post-treatment assessment, caregivers were administered the VABS-3 caregiver interview (Sparrow et al., 2016) to assess DLS. The VABS-3 DLS domain standard score (M = 100, SD = 15) was the primary outcome measures in the two recent RCTs completed on the STRW intervention. A score was calculated to assess change on the VABS-3 DLS domain standard score from baseline to post-treatment.

#### Behavior Assessment System for Children, 3^rd^ Edition (BASC-3).

At baseline, caregivers completed the BASC-3 (Kamphaus, 2015) to assess the teen’s behavior using the Internalizing Problems, Externalizing Problems, Behavioral Symptoms Index, and Adaptive Skills composite scale T-scores (M=50, SD=10).

#### BASC-3 Parenting Relationship Questionnaire (PRQ).

Caregivers completed the PRQ (Kamphaus & Reynolds, 2015) at baseline to capture the caregiver’s perspective on the relationship with their adolescent. The scales on the PRQ include Attachment (e.g., understanding teen’s thoughts and emotions, comforting teen), Communication (e.g., talking to and listening to the teen, talking about daily events with the teen), Discipline Practices (e.g., responding to misbehavior, establishing household expectations and rules), Involvement (e.g., planning and participating in activities together), Parenting Confidence (e.g., making parenting decisions), Satisfaction with School (e.g., feelings about whether school is meeting the teen’s needs), and Relational Frustration (e.g., reacting to common parenting situations such as arguments). All scales yield T-scores (M=50, SD=10).

### Analysis plan

Descriptive statistics were generated for the assessment data for participants at baseline and post-treatment. Paired sample t-tests were used to examine the profile of FQOL at baseline. Bivariate correlations were conducted to assess the relationship between FQOL and demographics, autism symptomatology, cognitive abilities, adaptive behavior, internalizing and externalizing behaviors, and parenting relationships. Paired sample t-tests were used to examine whether the FQOL subscales changed from baseline to post-treatment. Bivariate correlations were used to examine the relationship between FQOL at baseline and change in DLS on the VABS-3 from baseline to post-treatment. Statistical significance was set at *p* < .05. All analyses were conducted using SPSS version 29.

## Results

While a total of 60 participants completed the STRW intervention, only 58 participants completed the FQOL Scale at baseline. Further, only 48 of these participants also completed the FQOL Scale at post-treatment. There were no significant differences (all *p* values > 0.14) on FQOL at baseline between caregivers who completed the measure at post-treatment (n = 48) and those that did not (n = 10). Adolescent participants ranged from 14–18 years of age. While not reported here, participants who received the STRW intervention made significant gains on the VABS-3 DLS domain and improved from a mean standard score of 60.8 (17.9) at baseline to 79.4 (15.3) at post-treatment (Duncan et al., 2023; Duncan et al., in-press). See Table 1 for participant characteristics at baseline, including the means and standard deviations for all assessment measures.

### What is the profile of FQOL in caregivers of autistic teens?

See Table 2 for means and standard deviations of the subscale and overall score at baseline and post-treatment on the FQOL Scale. Results revealed the following pattern at baseline: Physical and Material Well-Being > Family Interaction > Parenting > Disability Related Support > Emotional Well-Being. Results of pair-sampled t-tests revealed that the Physical and Materials Well-Being mean item score was significantly greater than the Family Interaction (*p* < .001), Parenting (*p* < .001), Emotional Well-Being (*p* < .001), and Disability Related Support scores (*p* < .001); and that the Emotional Well-Being mean item score was significantly lower than the Family Interaction, Parenting, and Disability Related Support mean item scores (all *p* values <.001). No other significant differences between FQOL subscale scores were found (all *p* values >.08).

### What variables are associated with FQOL?

#### Demographics.

Only race was significantly correlated with FQOL (r = −.28, *p* = .04). Follow-up analyses revealed that caregivers who identified as Asian had significantly lower FQOL than caregivers who identified as White, Black, and More than One Race or Other (all *p* values <.03). However, this should be interpreted with caution because only 3 participants identified as Asian. Age, sex, ethnicity, education, and income were not significantly correlated with FQOL (all *p* values >.28).

#### Teen characteristics.

The SRS-2 Restricted Interests and Repetitive Behavior (r = −.30, *p* = .02) and Total scales (r = −.27, *p* = .04) were significantly correlated with FQOL. Cognitive ability (*p* = .64), the comparison score on the ADOS-2 (*p* = .17), the VABS-3 domains (all *p* values >.27), and the BASC-3 Composite Scales (all *p* values >.12) were not significantly correlated with FQOL.

#### Parenting characteristics.

FQOL was significantly correlated with the BASC-3 PRQ scales of Attachment (r = .35, *p* = .009), Discipline (r = .29, *p* = .03), Involvement (r = .37, *p* = .005), Parenting Confidence (r = .40, *p* = .002), School Satisfaction (r = .39, *p* = .002), and Relation Frustration (r = −.28, *p* = .04). The PRQ Communication scale was not significantly correlated (*p* = .09).

### Does a DLS intervention improve FQOL?

Results of paired sample t-tests revealed that the FQOL Family Interaction (*p* = .02) and Parenting (*p* = .03) subscales and the Overall score (*p* = .02) significantly improved from baseline to post-treatment (see Table 3). Changes in the Emotional Well-Being, Physical/Material Well-Being, and Disability Related Support subscales from baseline to post-treatment were not statistically significant (all *p* values >.13). Of note, there were no differences in FQOL at baseline or post-treatment between caregivers who received STRW in-person and caregivers who received it via telehealth (all *p* values >.21).

### Is FQOL linked to change in DLS after receiving the STRW intervention?

Bivariate correlations revealed a significant relationship between the FQOL Scale’s Emotional Well-Being subscale at baseline and the change in the VABS-3 DLS domain from baseline to post-treatment (r = .29, *p* = 03). There were no other significant correlations between FQOL and change on the VABS-3 DLS domain (all *p* values >.70).

## Discussion

This is the only known study that has examined FQOL in the caregivers of autistic adolescents without ID prior to their transition from high school to adulthood. On the Beach FQOL Scale, caregivers reported a profile that is consistent with what other studies have found in the caregivers of children, adolescents, and young adults with intellectual and developmental disabilities (Boehm et al., 2015; Hsiao et al., 2017): Physical and Material Well-Being > Family Interaction > Parenting > Disability Related Support > Emotional Well-Being. Caregivers of autistic teens in our study reported that the Physical and Material Well Being subscale (e.g., ability to take care of expenses, feeling of safety, and access to transportation and health care) was significantly higher than every other subscale. Conversely, the Emotional Well Being subscale (e.g., support to relieve stress, time to pursue own interests, access to supportive friends or family members, access to outside help to take care of special needs) was significantly lower than all of the other subscales. The results suggest that emotional well-being of caregivers may be an area of difficulty for families of autistic teens and a particularly important area to target in interventions to help support the entire family system.

The current study did not find that FQOL was correlated with demographic factors, which has been found in a previous study by Hsiao and colleagues (2017). However, importantly the study completed by Hsiao and colleagues (2017) had a larger and more diverse sample, which included a greater percentage of individuals with income below $70,000. It is possible that given we had a less diverse sample (i.e., majority white caregivers, greater percentage of higher income individuals, and mostly mothers), it may have limited our ability to detect meaningful differences in FQOL based on demographic factors. Additionally, there were no correlations between FQOL and cognitive ability, autism severity, adaptive behavior, and externalizing and internalizing symptoms in our sample. The correlation between FQOL and cognitive ability may be less pronounced in a sample of individuals without ID versus in a broader sample in which both individuals with and without ID were included. Lastly, it is possible that as autistic children move into adolescence and adulthood, other factors (e.g., coping, social support) may have greater influences on family quality of life compared to the factors outlined above (see Wang et al., 2022). Overall, more research is needed to identify and explore the predictors of FQOL in both autistic adolescents and their caregivers.

It is important to note that caregivers did report lower levels of FQOL if their teen had higher scores on the SRS-2 Restricted Interests and Repetitive Behavior Scale and Total Scale. Teens with rigid or inflexible behaviors or sensory interests and aversions may affect aspects of FQOL including how much time family members are able to spend together or how caregivers are able to assist with taking care of the teen’s needs (e.g., independence, social interactions, schoolwork). Further, these restricted interests and repetitive behaviors may impact the number or intensity of supports that may be needed to support the teen in various environments, which may increase stress in caregivers. In line with these findings, research has shown that increased child restricted and repetitive behaviors relates to increased caregiver stress (Harrop et al., 2016).

The FQOL Scale had significant, positive correlations with several BASC-3 PRQ scales including Attachment, Discipline, Involvement, Parenting Confidence, and School Satisfaction. This finding makes intuitive sense such that caregivers have increased levels of FQOL if they feel attached to their teen, involved in their teen’s life in the way that they want to be, capable of disciplining their teen, and confident in how they need to parent their teen. Further, if they are satisfied with the services and supports that they are receiving from their teen’s school, they are more likely to have increased rates of FQOL. These findings are in line with studies showing that caregiver of autistic youth with higher self-efficacy and greater social support reported higher FQOL (Dai et al., 2024) and that family-teacher partnerships have a direct effect on FQOL (Hsiao et al., 2017). Caregivers also reported that as relational frustration decreased with their teen, FQOL increased. These findings suggest that caregivers of autistic adolescents may benefit from an intervention that addresses how to navigate parenting their teen (e.g., how to handle conflict, how to set boundaries, how to connect with their teen, etc.) to increase overall FQOL.

Caregivers who participated in the STRW intervention endorsed significant changes on the Family Interaction and Parenting subscales and the Overall FQOL score from baseline to post-treatment. Items on the Family Interaction subscale include “my family solves problems together” and “my family members support each other to accomplish goals.” In STRW sessions, caregivers and teens were given the opportunity to engage in problem solving together as they worked towards identifying DLS goals. Therapists also provided individualized coaching or modeling on how to solve problems or achieve DLS goals. Similarly, an item on the Parenting subscale includes “family members help the children learn to be independent,” which is one of the main goals of the STRW intervention. It seems likely that the emphasis on building independence by having the caregiver and teen collaborate on developing a system of evidence based strategies to achieve DLS goals may positively affect FQOL. Further, additional elements of the intervention, such as the opportunity to get emotional support from other caregivers, may have led to positive changes in FQOL, which is in line with previous studies (Catalano et al., 2018; DaWalt et al., 2018).

A higher Emotional Well-Being subscale at baseline was the only FQOL variable that was related to increased changes in DLS from baseline to post-treatment after completing the STRW intervention. This suggests that families who have ways to relieve stress, have a support system, and have time to pursue their own interests may be better able to support adolescents as they work towards DLS goals and strive to build independence and autonomy. This speaks to the importance of clinicians who are delivering the STRW intervention to be aware of how families can utilize their current support systems (e.g., have a partner assist with teaching specific DLS) to help with achieving DLS goals. Further, therapists should discuss and highlight how building the teen’s independence may further reduce stress within the family (e.g., the teen learning how to cook dinner on evenings when the caregiver is working late). The STRW intervention has been recently refined to assist therapists with building their understanding of the family dynamic and ensuring that caregivers feel supported as they set realistic DLS goals with their teen. For example, at the end of each session, therapists specifically ask caregivers to reflect on whether the DLS goals for their teen are doable based on factors such as time and energy. Therapists are also provided with numerous examples of how they could incorporate DLS goals into the caregiver’s daily rhythm (e.g., have the teen help prepare dinner alongside the caregiver) or weekly rhythm (e.g., have the teen assist with loading the washing machine whenever the caregiver does laundry). These additions may help address emotional well-being while building the teen’s DLS. However, it is worth considering whether interventions targeting emotional well-being specifically may be beneficial for caregivers to complete concurrently or prior to starting STRW, especially for families for which this is an identified area of difficulty.

Taken together, the current findings highlight the importance of considering not only the characteristics of the autistic teen when providing treatment, but also considering the characteristics of the caregiver and the family system. Many interventions delivered to autistic adolescents rely on the caregiver to provide instruction, modeling, and coaching, and as such it is essential to understand how factors such as their emotional well-being and access to needed services at school and in the community may affect the ability to successfully commit to and implement the treatment plan. Clinicians may want to assess FQOL and other parenting factors (e.g., relationship with teen, parenting confidence) so that they can incorporate this when delivering treatment (e.g., discussing how to find ways to spend quality time with their teen). Further, if delivering a group intervention, it may be particularly powerful to discuss and normalize some of the parenting challenges that arise for caregivers of autistic adolescents. Lastly, it is promising to see that the STRW intervention not only leads to gains in age appropriate DLS in the adolescent, but also positive outcomes for the entire family system.

The current study does have several limitations. The various outcome measures and assessments used caregiver report of adolescent characteristics and family factors. Future studies should include the adolescent’s perspective on FQOL, and also explore how self-report on factors such as anxiety, depression, executive functioning, relationship with their caregiver, and self-determination may affect FQOL. Additionally, the sample lacked diversity in terms of race and ethnicity, socioeconomic status, and caregiver education, and the sample included primarily mothers. It is important to interpret these results within the context of the sample, as results may change given different participant characteristics. For instance, Physical/Material well-being may be significantly lower in samples that are experiencing higher levels of stress related to basic needs, and thus this area may need to be a greater treatment priority in different populations. Additionally, research has demonstrated that different family structures in which a grandparent or another extended family member serves as the primary caregiver may have a substantial impact on factors relevant to FQOL (e.g., Adams et al., 2025; Wu et al., 2017). Thus, results from the present study may not be generalizable to this population. It is imperative that future studies assess this construct in a more diverse set of caregiver participants to determine if the results and conclusions are applicable to more diverse samples. Finally, the mechanisms through which the STRW intervention positively influenced FQOL were not specifically assessed in this study. It would be informative to measure and better understand the factors from the intervention that specifically led to improvements in FQOL (e.g., getting emotional support from other caregivers, problem-solving difficulties that arose with a therapist, seeing gains in DLS, or increased confidence about the ability to set and achieve DLS goals in the future), so that these areas could be better targeted in future interventions.

### Overall conclusions and implications

FQOL may play a particularly critical role for caregivers of autistic teens and should be considered by clinicians delivering both individual and group treatment. For example, by addressing the emotional well-being of caregivers (e.g., identify stressors, discuss coping strategies, normalize teen’s challenges), it may not only increase the quality of life within the family environment, but may also lead to improved treatment gains in the teen.

## Figures and Tables

**Table 1:** Participant characteristics at baseline.

	STRW (n = 58)
Age	16.5 (1.2)
IQ	96.0 (10.1)
Sex at birth	
Male	48 (82.8%)
Female	10 (17.2%)
Race	
White	48 (82.8%)
Black	4 (6.9%)
Asian	3 (5.2%)
Other or More Than One Race	3 (5.2%)
Ethnicity	
Hispanic	2 (3.4%)
Non-Hispanic	56 (96.6%)
Yearly Household Income	
Less than $20,000	2 (3.4%)
$20,000 – $50,000	4 (6.9%)
$50,001 – $80,000	11 (19.0%)
$80,001 – $100,000	9 (15.5%)
$100,001 – $130,000	14 (24.1%)
Over $130,000	16 (27.5%)
Not reported	2 (3.4%)
VABS–3	
Communication Domain Standard Score	67.5 (13.0)
DLS Domain Standard Score	61.1 (17.7)
Socialization Domain Standard Score	65.3 (12.4)
ABC Domain Standard Score	64.6 (10.3)
SRS– 2	
Social Awareness T-Score	69.9 (9.3)
Social Cognition T-Score	71.1 (9.2)
Social Communication T-Score	73.0 (9.6)
Social Motivation T-Score	72.1 (12.3)
Restricted Interests and Repetitive Behavior T-Score	74.2 (11.1)
Total T-Score	75.1 (9.2)
BASC–3	
Externalizing Problems T-Score	55.3 (10.1)
Internalizing Problems T-Score	56.4 (11.6)
Behavioral Symptoms Index T-Score	62.3 (8.7)
Adaptive Skills T-Score	37.6 (6.7)
BASC PRQ	
Attachment T-Score	46.1 (10.3)
Communication T-Score	41.5 (9.8)
Discipline Practices T-Score	43.7 (7.1)
Involvement T-Score	46.9 (8.6)
Parenting Confidence T-Score	44.1 (11.7)
Satisfaction with School T-Score	47.4 (9.6)
Relational Frustration T-Score	55.4 (9.8)

**Table 2:** FQOL Scale Mean Item Score at Baseline (n = 58).

Subscale	M (SD)
Parenting	4.07 (0.68)
Family Interaction	4.00 (0.56)
Emotional Well Being	3.54 (0.84)
Physical/Material Well-Being	4.48 (0.53)
Disability-Related Support	3.92 (0.82)
Total	4.03 (0.55)

* Scores range from 1–5 with a higher score being indicative of increased satisfaction.

**Table 3:** Change in FQOL Subscale Mean Item Score from Baseline to Post-Treatment (n = 48).

Subscale	Score at Baseline M (SD)	Score at Post-treatment M (SD)	*p*
Parenting	4.01 (0.57)	4.16 (0.70)	.02
Family Interaction	4.10 (0.66)	4.25 (0.71)	.03
Emotional Well Being	3.61 (0.81)	3.72 (0.81)	.12
Physical/Material Well-Being	4.52 (0.48)	4.60 (0.42)	.09
Disability-Related Support	3.96 (0.85)	4.07 (0.80)	.13
Total	4.06 (0.56)	4.18 (0.58)	.02

* Scores range from 1–5 with a higher score being indicative of increased satisfaction.

## Data Availability

Data will be made available on request. Data is available from the first author upon reasonable request.
